# Situational confidence and recovery capital among justice-involved adults receiving medications for opioid use disorder in a jail-based setting

**DOI:** 10.1186/s40352-025-00377-x

**Published:** 2025-11-04

**Authors:** Elizabeth O. Obekpa, Xulei He, Alejandro Betancur, Kathryn R. Gallardo, Serena A. Rodriguez, Sheryl A. McCurdy, J. Michael Wilkerson

**Affiliations:** School of Public Health, The University of Texas Health Science Center at Houston, Texas Houston, United States

**Keywords:** Situational confidence, Recovery capital, Opioid use disorder, MOUD, Justice-involved, Jail-based treatment, Relapse prevention

## Abstract

**Background:**

Justice-involved individuals with opioid use disorder (OUD) face heightened relapse risks during the reentry period. While medications for opioid use disorder (MOUD) are effective, long-term recovery also depends on psychological and structural supports, including situational confidence (perceived ability to resist substance use in high-risk situations) and recovery capital (the internal and external resources that support recovery).

**Methods:**

This cross-sectional study examined situational confidence and its association with recovery capital among 107 justice-involved adults receiving MOUD in an urban jail in Texas. Participants completed the Brief Situational Confidence Questionnaire (BSCQ) and the 10-item Brief Assessment of Recovery Capital (BARC-10). Mental health and substance use were assessed using the PHQ-9, GAD-7, AUDIT, and ASSIST. Bivariate associations were examined using chi-square and Fisher’s exact tests, and a multivariable logistic regression model was used to assess factors associated with high situational confidence (BSCQ ≥ 80%).

**Results:**

Participants had a mean age of 38.9 years (SD = 0.4); most were male (74.0%), non-Hispanic Black or other race/ethnicity (58.0%), and had a high school education or less (59.8%). A majority (66.4%) reported unstable housing in the 30 days prior to incarceration. Fewer than half (44.9%) reported high situational confidence, with a mean score of 67.6 (SD = 26.9). Higher recovery capital was strongly associated with high situational confidence (aOR = 2.66; 95% CI: 1.52–4.96). Depression (aOR = 0.36; 95% CI: 0.16–0.78), sexual minority status (aOR = 0.14; 95% CI: 0.01–0.78), and reliance on informal income-generating activities (“hustling”) (aOR = 0.30; 95% CI: 0.10–1.01) were associated with lower situational confidence.

**Conclusions:**

Recovery capital is a strong predictor of situational confidence among justice-involved individuals receiving MOUD. Interventions that enhance recovery capital, including access to employment, housing, and social support, integrate mental health care, and provide tailored support for marginalized subgroups, may improve recovery outcomes during incarceration and reentry. Brief, validated tools like the BSCQ and BARC-10 may help identify individuals at greater relapse risk and guide more targeted, equity-informed reentry planning.

## Introduction

The opioid epidemic remains a critical public health crisis in the United States, with an estimated 5.6 million individuals meeting diagnostic criteria for opioid use disorder (OUD) in 2021 (Substance Abuse and Mental Health Services Administration (SAMHSA), 2022). Justice-involved individuals are disproportionately affected by OUD and are 8 to 10 times more likely to meet diagnostic criteria than the general population (Wakeman et al., 2023; Winkelman et al., 2022). The risk of overdose death is particularly acute during the first two weeks following release, when it increases 10- to 40-fold (Binswanger et al., 2007; Hartung et al., 2023; Marsden et al., 2017).

Justice-involved populations often face overlapping vulnerabilities that compound barriers to treatment and recovery, including sexual and gender minority (SGM) status, low socioeconomic status, co-occurring mental health disorders, and polysubstance use. SGM individuals often experience stigma and limited access to affirming care (Coulter et al., 2019; Salcedo-Betancourt et al., 2022), while those with low socioeconomic status face housing instability, unemployment, and limited healthcare access (Dasgupta et al., 2018). Mental health conditions like depression and anxiety can undermine treatment adherence (Baxley et al., 2019; Sullivan, 2018), and concurrent use of substances such as alcohol or methamphetamine increases overdose risk (Korthuis et al., 2022; Winstanley et al., 2020). These intersecting challenges elevate the risk of recurrence and underscore the need for recovery supports that address both clinical and structural vulnerabilities.

Medications for OUD (MOUD), including methadone and buprenorphine, are effective in reducing withdrawal symptoms, preventing overdose, and improving treatment retention (National Institute on Drug Abuse [NIDA], 2022); SAMHSA, 2023). MOUD also significantly reduces overdose risk, recidivism, and post-release opioid use among justice-involved individuals with OUD (Grella et al., 2020). However, medication alone may be insufficient for sustained recovery, particularly among individuals with complex psychosocial needs. Two key concepts in addiction recovery—recovery capital and situational confidence—are increasingly recognized as critical for long-term abstinence and resilience (Britton et al., 2023; Cano et al., 2017; Laudet & White, 2008; O’Sullivan, 2016).

Recovery capital, defined as the breadth of internal and external assets that support recovery, offers a valuable framework for understanding resilience in individuals with substance use disorders (Cloud & Granfield, 2008; White & Cloud, 2008). It includes human, social, physical, cultural, and community domains (Hennessy, 2020). Emerging evidence shows that recovery capital can increase during and after incarceration through structured, continuity-focused interventions that include peer support and recovery housing (Best et al., 2024).

Situational confidence, rooted in Bandura’s (1977) self-efficacy theory, refers to one’s perceived ability to resist substance use in high-risk situations, which may include triggers such as negative affect, cravings, or social pressure (Annis & Graham, 1988; Breslin et al., 2000). Situational confidence is consistently associated with reduced relapse and improved recovery outcomes (Al-Ziadat, 2024; Ilgen et al., 2005a; Kadden & Litt, 2011). Research suggests recovery capital predicts situational confidence in community-based samples (Gilbert & Kurz, 2018; Obekpa et al., 2023; O’Sullivan et al., 2016), but this relationship has not been examined in correctional settings.

Justice-involved individuals face unique structural barriers during reentry, including stigma, disrupted social networks, and limited access to employment and supportive services (Binswanger et al., 2012; Kahn et al., 2019). These factors may differentially impact both recovery capital and confidence in managing substance use triggers. Understanding how these constructs interact in jail-based settings could inform more comprehensive interventions that enhance not only pharmacologic treatment but also the psychological and structural resources needed to sustain abstinence after release.

This study aims to assess levels of situational confidence and examine its relationship with recovery capital among individuals receiving MOUD in a large urban jail. We also explore associations with mental health symptoms, substance use severity, and sociodemographic characteristics.

## Methods

### Study design

Incarcerated individuals (*N* = 107) in a metropolitan area in southeast Texas completed a cross-sectional survey as part of the Centers for Disease Control and Prevention’s Opioid Data to Action Project (ODTA) (CDC-RFA-CE19-1904), Strategy 6: Linkage to Care. The UTHealth Houston Committee for the Protection of Human Subjects approved the study protocol, survey, and informed consent form under IRB number HSC-SPH-20–1385.

### Sampling, recruitment, and procedures

Participants were 18 + years old, spoke English or Spanish, resided in the seven-county metropolitan area after release, had a sentence that did not exceed the time of the project, and were diagnosed with alcohol and/or opioid use disorder. Licensed chemical dependency counselors screened and diagnosed potential participants and referred them to the project. Eligible participants received peer recovery coaching with a certified recovery coach and individual counseling with a licensed chemical dependency counselor while incarcerated. Participants received a post-discharge referral list of services upon release and a 2-month, extended-release injectable naltrexone prescription referral post-release. Participants completed an interviewer-administered survey inside the jail, and responses were saved on REDCap. The structured interviews lasted between 90 and 120 min. Participants were compensated $25 in cash upon release.

### Measures

#### Sociodemographic characteristics

Participants self-reported their age, gender identity, race/ethnicity, sexual orientation, marital status, education, employment status, and housing stability in the 30 days prior to incarceration. Employment status was categorized as: (1) employed or enrolled as a student, without hustling (defined as informal or illicit income), (2) unemployed, retired, or residing in a controlled environment without hustling, and (3) engaged in hustling. Housing was classified as stable (e.g., owned or rented home, group home, veteran’s home, or permanent single-room occupancy [SRO]) or unstable (e.g., homelessness, shelters, temporary SROs, hospitals, treatment facilities, or temporarily staying with others).

#### Situational confidence

The 8-item Brief Situational Confidence Questionnaire (BSCQ; Breslin et al., 2000) assessed participants’ perceived ability to resist substance use across eight high-risk scenarios. Items were rated from 0% (“not at all confident”) to 100% (“completely confident”). A mean score was calculated, with scores dichotomized into low-to-moderate confidence (< 80%) and high confidence (≥ 80%), following Obekpa et al. (2023).

#### Recovery capital

Recovery capital was measured using the 10-item Brief Assessment of Recovery Capital (BARC-10), with items rated on a 6-point Likert scale from “strongly disagree” to “strongly agree.” Higher total scores indicate greater recovery capital (Vilsaint et al., 2017).

#### Depression and anxiety

Depression and anxiety symptoms were assessed using the Patient Health Questionnaire-9 (PHQ-9) and Generalized Anxiety Disorder-7 (GAD-7), respectively. Both scales evaluate symptom frequency over the past two weeks on a 4-point scale (0 = “not at all” to 3 = “nearly every day”). Standard cutoff scores (≥ 10) were used to define moderate or greater symptom severity (Kroenke et al., 2001; Spitzer et al., 2006).

#### Substance use severity

Substance use was assessed with the Alcohol, Smoking, and Substance Involvement Screening Test (ASSIST), which measures lifetime and recent use of various substances (WHO ASSIST Working Group, 2002). Higher scores reflect greater involvement. Alcohol-specific risk was further evaluated using the 10-item Alcohol Use Disorders Identification Test (AUDIT), with scores categorized as low/moderate risk (0–15), high risk (16–19), and probable alcohol dependence (≥ 20) (Babor et al., 2001).

### Data analysis

Descriptive statistics were calculated for all sociodemographic variables and measures of mental health, substance use, and recovery-related variables. Participants were grouped by situational confidence level (low-to-moderate vs. high), and group differences were assessed using t-tests for continuous variables and chi-square or Fisher’s exact tests for categorical variables, with statistical significance set at *p* < 0.10. Variables that differed significantly between groups were entered into multivariable logistic regression models to identify predictors of high situational confidence (*p* < 0.05).

## Results

### Participant characteristics

Table 1 presents descriptive statistics for the study sample. A total of 107 justice-involved adults receiving MOUD in a large urban jail participated in the study. Participants had a mean age of 38.9 years (SD = 0.4). Most were male (74.0%), identified as non-Hispanic Black or other race/ethnicity (58.0%), and had a high school diploma or less (60.0%). Prior to incarceration, 53.0% were employed or students, 28.0% were unemployed, and 17.0% reported “hustling” as their primary source of income. Approximately 8.4% identified as sexual minorities, and no participants identified as transgender or non-binary. Housing instability was common, with 66.4% reporting unstable housing in the 30 days prior to incarceration.

Mental health symptoms were also prevalent: 51.4% screened positive for moderate to severe depression (PHQ-9 score ≥ 10), and 55.1% met the threshold for anxiety (GAD-7 score ≥ 10). In terms of alcohol use severity, 28.0% were categorized as low to moderate risk, 4.7% as high risk, and 52.3% met the criteria for alcohol dependence based on AUDIT scores. The mean ASSIST score was 17.3 (SD = 0.4), indicating moderate substance use severity.

At the bivariate level, situational confidence was significantly associated with sexual minority status (*p* = 0.04), employment status (*p* = 0.08), depression (*p* = 0.01), alcohol use severity (*p* = 0.03), and recovery capital (*p* < 0.001). No significant associations were observed for age, gender, race/ethnicity, education, marital status, housing stability, anxiety, or overall substance use severity.

**Table 1:** Participant characteristics and bivariate associations with situational confidence (*N* = 107)

Characteristics	Overall *n* (%)	Situational Confidence		*P*-value
		Low-Moderate (< 80%) *n* = 59 (55.1%)	High (*≥* 80%) *n* = 48 (44.9%)	
Age (mean [SD])	38.9 [0.4]	40.1 [0.4]	37.4 [0.4]	0.21
Gender				0.52
Woman	28.0 (26.0%)	17.0 (29.0%)	11.0 (23.0%)	
Man	79.0 (74.0%)	42.0 (71.0%)	37.0 (77.0%)	
Sexual minority				**0.04**
Yes	9.0 (8.4%)	8.0 (14.0%)	1.0 (2.0%)	
No	98.0 (92.0%)	51.0 (86.0%)	47.0 (98.0%)	
Race-ethnicity				0.65
Hispanic	25.0 (23.0%)	14.0 (24.0%)	11.0 (23.0%)	
Non-Hispanic white	20.0 (19.0%)	11.0 (19.0%)	9.0 (19.0%)	
Non-Hispanic Black/Other	44.0 (58.0%)	23.0 (39.0%)	21.0 (44.0%)	
Education				0.64
High school or less	64.0 (60.0%)	36.0 (61.0%)	28.0 (58.0%)	
Some college/vocational-technical/college	26.0 (24.0%)	13.0 (22.0%)	13.0 (27.0%)	
Employment				**0.08**
Employed or student, no hustling	57.0 (53.0%)	29.0 (49.0%)	28.0 (58.0%)	
Unemployed, retired, in a controlled environment, no hustling	30.0 (28.0%)	14.0 (24.0%)	16.0 (33.0%)	
Hustling	18.0 (17.0%)	14.0 (24.0%)	4.0 (8.0%)	
Marital status				0.46
Married or common law	21.0 (20.0%)	9.0 (15.0%)	12.0 (25.0%)	
Single/Never married	51.0 (48.0%)	29.0 (49.0%)	22.0 (46.0%)	
Divorced, separated, or widowed	18.0 (17.0%)	11.0 (19.0%)	7.0 (15.0%)	
Housing Stability 30 days prior to incarceration				0.54
Stable housing	36.0 (34.0%)	18.0 (31.0%)	18.0 (38.0%)	
Unstable housing	71.0 (66.0%)	41.0 (69.0%)	30.0 (63.0%)	
PHQ-9 (0–27)				**0.01**
No depression (< 10)	52.0 (49.0%)	22.0 (37.0%)	30.0 (63.0%)	
Depression (*≥* 10)	55.0 (51.0%)	37.0 (63.0%)	18.0 (38.0%)	
GAD-7 (0–21)				0.12
No anxiety (< 10)	48.0 (45.0%)	22.0 (37.0%)	26.0 (54.0%)	
Anxiety (> 10)	59.0 (55.0%)	37.0 (63.0%)	22.0 (46.0%)	
ASSIST (mean [SD])	17.3 [0.4]	18.3 [0.4]	16.1 [0.3]	0.15
AUDIT				**0.03**
Low/moderate risk (0–15)	30.0 (28.0%)	12.0 (20.0%)	18.0 (38.0%)	
High risk (16–19)	5.0 (5.0%)	5.0 (8.0%)	0.0 (0.0%)	
Alcohol dependence (> 20)	56.0 (52.0%)	32.0 (54.0%)	24.0 (50.0%)	
BARC (mean [SD])	4.83 [0.8]	4.60 [0.8]	5.12 [0.7]	**< 0.01**

*SD* Standard deviation, Boldface indicates significant at *p* < 0.1

Table 2 summarizes participants’ scores on situational confidence and recovery capital measures. The mean situational confidence score was 67.6 (SD = 26.9), with a minimum of 0 and a maximum of 100. Less than half (44.9%) of participants reported high situational confidence (defined as BSCQ ≥ 80%). The mean recovery capital score, measured by the BARC-10, was 4.8 (SD = 0.8), with scores ranging from 2.2 to 6.0. Participants with high situational confidence had significantly higher BARC-10 scores (mean = 5.1, SD = 0.7) than those with low-to-moderate confidence (mean = 4.6, SD = 0.8), *p* < 0.01.

**Table 2:** Participants’ scores on situational confidence and recovery capital measures

Variable	Mean	Standard Deviation	Scale Range	Min	Max
Situational Confidence (BSCQ)	67.6	26.9	0–100	0	100
Recovery Capital (BARC-10)	4.8	0.8	1–6	2.2	6.0

Min and Max represent the lowest and highest observed scores in the sample for each measure

**Fig. 1:**
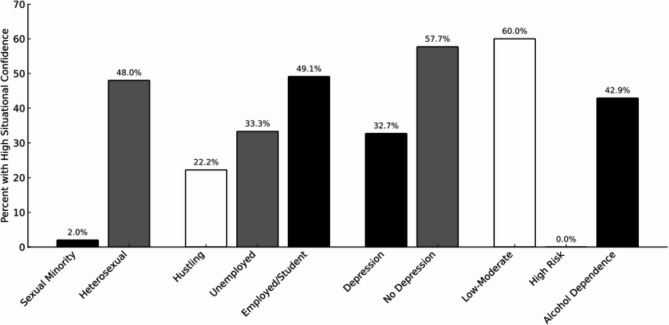
Percentage of participants reporting high situational confidence (≥80%)

Figure 1 illustrates key group-level differences. Only 2.0% of sexual minority participants reported high situational confidence, compared to 48.0% of heterosexual participants. Among employment groups, individuals engaged in hustling had low situational confidence (22.2%), while 49.1% of employed participants or those in school had high confidence. Depression was associated with significant differences in situational confidence, with 57.7% of participants without depressive symptoms reporting high confidence compared to 32.7% of participants with symptoms. A similar pattern was observed across alcohol risk categories, with 60.0% of low-to-moderate risk participants reporting high situational confidence, followed by 42.9% of those with alcohol dependence. No high-risk drinkers reported high situational confidence.

Table 3 presents the results of the multivariable logistic regression analysis examining factors associated with high situational confidence. After adjusting for other variables, participants who reported hustling as their primary income source had significantly lower odds of high situational confidence compared to those who were employed or students (aOR = 0.30; 95% CI: 0.10, 1.01). Sexual minority participants also had lower odds of high situational confidence compared to non-minority participants (aOR = 0.14; 95% CI: 0.01, 0.78). Depression was significantly associated with lower situational confidence; individuals with depressive symptoms had 64% lower odds of reporting high confidence (aOR = 0.36; 95% CI: 0.16, 0.78). Recovery capital remained a strong independent predictor of high situational confidence, with each one-point increase in BARC-10 score associated with more than twice the odds of high confidence (aOR = 2.66; 95% CI: 1.52, 4.96; *p* = 0.001). The high-risk alcohol use group (AUDIT scores 16–19) was excluded from the multivariate analysis due to low cell count. Alcohol dependence was associated with lower odds of high confidence, although this association did not reach statistical significance (aOR = 0.50; 95% CI: 0.20, 1.22).

**Table 3:** Multivariable logistic regression of factors associated with high situational confidence

High situational confidence	aOR [95% CI]
Employment	
Employed or student, no hustling	REF
Unemployed, retired, in a controlled env., no hustling	1.18 [0.49, 2.87]
Hustling	**0.30 [0.10**,**1.00]**
Sexual minority	
No	REF
Yes	**0.14 [0.01**,** 0.78]**
PHQ-9 (0–27)	
No depression (< 10)	REF
Depression (> 10)	**0.36 [0.16**,** 0.78]**
AUDIT	
Low/moderate risk (0–15)	REF
Alcohol dependence (> 20)	0.50 [0.20, 1.22]
BARC	**2.66 [1.52**,** 4.96]**

*env* Environment, *aOR* Adjusted Odds Ratio; Boldface indicates significant at *p* ≤ 0.05

## Discussion

We found that recovery capital was the strongest predictor of situational confidence among justice-involved adults receiving MOUD, while fewer than half of the participants reported high confidence levels. Depression, sexual minority status, and engagement in informal income generation were associated with reduced confidence to resist substance use. These findings provide the first examination of this relationship in a U.S. jail-based setting and reveal important targets for intervention.

The mean situational confidence score of 67.6 was substantially lower than reported in community-based recovery settings, where participants typically score above 80 (Obekpa et al., 2023; O’Sullivan et al., 2016). These findings are particularly concerning given that maximal confidence (100%) offers the strongest protection against recurrence (Ilgen et al., 2005b), suggesting that moderate gains may be insufficient for sustained abstinence in this high-risk population. This disparity highlights the unique challenges faced by justice-involved individuals with OUD, whose confidence may be undermined by limited autonomy, systemic barriers such as stigma, interrupted access to MOUD during incarceration and reentry, and structural factors including unemployment, housing instability, inadequate social support, limited resources, and restricted access to healthcare (Grella et al., 2020; Strong‑Jones et al., 2024).

The high prevalence of low situational confidence in our sample indicates substantial vulnerability to recurrence during the reentry period, when justice-involved individuals face elevated relapse risk due to social and environmental triggers and limited access to recovery supports (Binswanger et al., 2012; Davis et al., 2022). Although participants received services from recovery support peer specialists and licensed chemical dependency counselors, existing jail-based interventions may lack the intensity or integration needed to effectively strengthen recovery capital before release. For individuals with OUD, these challenges are compounded by the physiological burden of opioid dependence, which can further erode confidence in resisting use (Wakeman & Rich, 2018).

Recovery capital emerged as the strongest predictor of high situational confidence, with each one-point increase in BARC-10 score more than doubling the odds of high confidence. This finding aligns with prior community-based research (Gilbert & Kurz, 2018; Obekpa et al., 2023; O’Sullivan, 2016), but extends the evidence to justice-involved individuals receiving MOUD in correctional settings for the first time. The strength of this association suggests that building recovery assets, such as social support, employment readiness, and coping skills, may be as critical as medication provision in preparing individuals for successful reentry and reducing reoccurrence risk during the vulnerable post-release period. Importantly, recovery capital is dynamic and can be strengthened through structured, recovery-oriented interventions, including therapeutic communities and educational programming (Best et al., 2024; Knapp et al., 2024).

Depression significantly predicted lower situational confidence, with over half of the participants screening positive for depressive symptoms. This is consistent with prior literature linking mental health disorders to impaired self-efficacy and increased risk of relapse (Greenfield et al., 2012). Our finding reinforces the need for integrated behavioral health care within MOUD programs, particularly screening and treatment for depression during pre-release planning.

Sexual minority status was also associated with lower situational confidence. This finding reflects broader disparities among SGM populations with substance use disorders, who often face stress, stigma, discrimination, limited socio-economic resources, physical and sexual assault, and high psychological distress, during and after incarceration (Harvey et al., 2022; Ogunbajo et al., 2023). Only 2.0% of sexual minority participants reported high confidence compared to 48.0% of heterosexual participants. However, the small number of sexual minority participants in our sample requires cautious interpretation of this finding, though it is consistent with broader literature documenting substance use disparities among SGM populations.

Engagement in “hustling” was also associated with lower confidence, likely reflecting exposure to drug-related environments and chaotic routines that undermine recovery motivation. This informal income generation may expose individuals to substance use triggers while reinforcing unstable daily patterns that conflict with recovery goals. Such economic marginalization creates a cycle where individuals lack both the financial stability and environmental support necessary for sustained abstinence. This finding emphasizes the importance of vocational training and legal income pathways in reentry programming, as employment is a known protective factor against relapse and is associated with higher levels of recovery capital among justice-involved individuals with OUD (Best et al., 2024).

### Practice, policy, and research implications

The study findings have several implications for practice, policy, and research. Clinically, brief tools such as the BSCQ and BARC-10 should be integrated into standard discharge planning to identify individuals at heightened relapse risk and guide personalized, recovery-oriented interventions. Jail-based MOUD programs should expand beyond medication provision to include structured supports that build recovery capital, such as peer coaching, educational programming, and discharge planning that addresses housing, employment readiness, and mental health needs. Given the strong association between depression and lower situational confidence, routine screening and treatment for co-occurring mental health disorders should be embedded within MOUD services. Programs must also prioritize culturally responsive care, particularly for sexual and gender minorities and socioeconomically marginalized individuals, through trauma-informed, affirming services that include legal advocacy and peer-based support.

Beyond individual-level interventions, broader system changes are needed. Continuity of care during the reentry period is essential. Warm handoffs to community providers, seamless transitions in medication access, and linkage to housing and employment programs are critical for sustaining recovery. Structural barriers, such as limited healthcare access, fragmented discharge planning, and weak community linkages, continue to undermine recovery efforts in carceral settings, particularly across the southern U.S. (Brinkley-Rubinstein et al., 2025; Rhodes Fortino et al., 2024). At the systems level, policy reforms should focus on expanding the use of Sect. 1115 Medicaid waivers to provide pre-release coverage and care coordination, as well as investing in wraparound reentry services. However, proposed federal changes to Medicaid policy could threaten the feasibility and sustainability of jail-based MOUD programs and reentry services, especially in under-resourced jurisdictions.

Future research should employ longitudinal designs to examine how recovery capital and situational confidence evolve across incarceration, reentry, and long-term recovery. Understanding the timing and durability of these constructs can inform the development of phased, adaptive interventions. Additionally, studies should explore whether different MOUD types or treatment durations influence recovery capital accumulation or situational confidence in this population. Research examining optimal intervention timing and subgroup-specific approaches could further refine recovery-oriented programming for diverse populations within correctional settings, as individuals may experience different relationships between recovery capital domains and self-efficacy at various stages of their recovery journey.

### Study limitations

This study has several limitations. Its cross-sectional design limits causal inference and does not capture the dynamic evolution of recovery capital over time. All measures were self-reported, which may introduce recall bias or social desirability effects. The sample, drawn from a single urban jail in Texas and limited to English- or Spanish-speaking adults with alcohol/OUD or residing in a specific seven-county metropolitan area, may limit generalizability to other regions, carceral settings, or populations with different demographic characteristics. The modest sample size limited the precision of subgroup comparisons, particularly for SGM participants, and requires cautious interpretation of our findings. We did not assess whether substance use occurred during incarceration, which may influence situational confidence. Additionally, we did not examine whether primary substance use patterns moderated the relationship between recovery capital and situational confidence, despite evidence that different recovery capital domains may vary in relevance for alcohol versus opioid use. Finally, although all participants received MOUD, we did not differentiate between methadone, buprenorphine, or naltrexone, or account for treatment duration, factors that may differentially influence both recovery capital and situational confidence.

## Conclusion

Recovery capital represents a critical but modifiable predictor of situational confidence among justice-involved individuals receiving MOUD. The substantially lower confidence levels in this population, compared to community settings, underscore the need for comprehensive, recovery-oriented interventions that address multiple domains of recovery capital alongside medication provision. Addressing employment and economic stability emerges as particularly important, given the strong association between informal income generation and reduced confidence in abstaining from substance use. Brief assessment tools can identify high-risk individuals and guide targeted reentry planning. Addressing mental health needs and providing culturally responsive care for vulnerable populations are essential components of effective jail-based MOUD programs. These findings provide an evidence base for transforming correctional substance use treatment from medication-focused to recovery-oriented systems of care that include vocational training and pathways to stable employment as core components of reentry preparation.

## Data Availability

The datasets generated and/or analysed during the current study are not publicly available due to ethical restrictions and participant confidentiality concerns, but are available from the corresponding author on reasonable request.
